# Integrative analyses of genetic characteristics associated with skeletal endothelial cells

**DOI:** 10.1590/1414-431X2024e13339

**Published:** 2024-04-19

**Authors:** Zhanhui Wang, Bowen Hu, Xiaoming Chen, Zheng Zhang, Lu Liu, Nan Li, Chun Liang

**Affiliations:** 1 Department of Cardiology, Second Affiliated Hospital of Naval Medical University, Shanghai, China; 2 Department I of Cadre's Ward, Navy 971st Hospital, Qingdao, China; 3 Department of Orthopedic Rehabilitation, Qingdao Special Servicemen Recuperation Center of PLA Navy, Qingdao, China

**Keywords:** Skeletal endothelial cells, Type H vessel, Bone, Osteoporosis, Bioinformatics

## Abstract

The osseous vascular endothelium encompasses a vast intricate framework that regulates bone remodeling. Osteoporosis, an age-associated systemic bone disease, is characterized by the degeneration of the vascular architecture. Nevertheless, the precise mechanisms underpinning the metamorphosis of endothelial cells (ECs) with advancing age remain predominantly enigmatic. In this study, we conducted a systematic analysis of differentially expressed genes (DEGs) and the associated pathways in juvenile and mature femoral ECs, utilizing data sourced from the Gene Expression Omnibus (GEO) repositories (GSE148804) and employing bioinformatics tools. Through this approach, we successfully discerned six pivotal genes, namely *Adamts1*, *Adamts2*, *Adamts4*, *Adamts14*, *Col5a1*, and *Col5a2*. Subsequently, we constructed a miRNA-mRNA network based on miRNAs displaying differential expression between CD31^hi^EMCN^hi^ and CD31^low^EMCN^low^ ECs, utilizing online repositories for prediction. The expression of miR-466i-3p and miR-466i-5p in bone marrow ECs exhibited an inverse correlation with age. Our *in vivo* experiments additionally unveiled miR-466i-5p as a pivotal regulator in osseous ECs and a promising therapeutic target for age-related osteoporosis.

## Introduction

Bone marrow and endosteum/periosteum are richly endowed with blood vessels, which form an intricately intertwined network that provides nutrients and oxygen and at the same time eliminates metabolic byproducts (1). Endothelial cells (ECs) are the main constituent of the vasculature and have a pivotal function in controlling vascularization (2). Within the adult osseous tissue, there are two distinct subtypes of blood vessels: type H, characterized by elevated expression levels of CD31 and endomucin (EMCN) within the endothelium, and type L, with relatively diminished expression levels of CD31 and EMCN within the endothelium (3). CD31^hi^Emcn^hi^ ECs are predominantly located in the vicinity of the growth plate or periosteal/endosteal surface, where they promote osteogenesis of skeletal stem cells through the secretion of paracrine molecules (4). In contrast, CD31^low^Emcn^low^ ECs are distributed within the bone marrow cavity (4). An abnormal skeletal vessel meshwork is implicated in the pathogenesis of multiple bone diseases (5).

Osteoporosis, a condition characterized by the systemic demineralization of bone and an increased susceptibility to fractures, has a strong correlation with age-related phenomena such as senescence, malnutrition, and menopause (6,7). With the growing elderly population, the prevalence of osteoporosis has increased in recent years. It has been estimated that globally, 23.1% of elderly women and 11.7% of elderly men endure diminished bone density or osteoporosis, culminating in high healthcare expenditures amounting to billions of dollars (8). The age-related deterioration of the osseous endothelium contributes to an imbalance between bone resorption and formation, serving as the fundamental cellular substrate for osteoporosis pathogenesis (9-11). Nevertheless, the genetic mechanisms of the transition of the skeletal endothelium remain mostly unknown.

MicroRNAs (miRNAs) are short, single-stranded, non-coding RNA molecules composed of 18-25 nucleotides (12). Their primary function is to negatively regulate gene expression patterns by binding to specific target mRNAs (13). For example, the miR-466i gene family exerts regulatory control over the processes of inflammation and apoptosis (14). To illustrate, within the context of heatstroke, miR-466i-5p has the ability to instigate cerebral damage by facilitating apoptotic events within the hippocampal neurons (15). Additionally, in the early stages of infection, antimony-resistant *Leishmania donovani* selectively binds to miR-466i to disable the host MyD88, thereby modulating the levels of interleukin (IL)-10 and IL-12 (16). Prior investigations have also documented that the establishment and functionality of type H vessels are under the regulatory influence of a repertoire of miRNAs (17). Nevertheless, the precise interplay among miRNAs, skeletal ECs, and the intricacies of bone metabolism needs further study.

In recent years, the advent of high-throughput sequencing technology and profound advances in bioinformatics have made it possible to study biological processes at the genetic level. In this investigation, we used the power of bioinformatics to examine genetic modifications occurring within bone ECs across diverse age groups, using data from the Gene Expression Omnibus (GEO) repositories. Our findings indicated a remarkable enrichment of osteogenic pathways in juvenile femoral ECs. Moreover, through the analysis of protein-protein interactions (PPI), we successfully identified six pivotal genes, namely *Adamts1*, *Adamts2*, *Adamts4*, *Adamts14*, *Col5a1*, and *Col5a2*. These genes hold substantial potential as diagnostic biomarkers and therapeutic targets for age-related bone loss.

## Material and Methods

### Sample collection

All gene expression datasets were obtained from GEO (https://www.ncbi.nlm.nih.gov/geo/). The investigation (GSE148804), based on GPL19057Illumina NextSeq 500 (Mus musculus), was conducted on capillaries from the ossification front of the juvenile (5 samples) and adult (4 samples) mouse femurs (18). The miRNA expression microarray GSE95196 included 6 samples of ECs (3 samples of type H ECs and 3 samples of type L ECs). This dataset was based on GPL21265 Agilent-070155 Mouse miRNA Microarray (miRBase Release 21.0, miRNA ID version) (17).

### Identification and functional analyses of DEGs

Differentially expressed genes (DEGs) were identified using the Limma package by comparing the mRNA profile of juvenile and adult mouse femoral capillaries (GSE148804). Moreover, differentially expressed miRNAs (DEmiRNAs) between type H and type L ECs were obtained by the Limma package (GSE95196; https://bioconductor.org/packages/release/bioc/html/limma.html). An adjusted P-value of 0.05 and a fold-change of 1 were set as criteria of DEGs.

The enrichment terms in GO and Kyoto Encyclopedia of Genes and Genomes (KEGG) were analyzed by ClusterProfiler package in R (Lucent Technologies, USA). The ggplot2 package (https://ggplot2.tidyverse.org/) was adopted to visualize the top 10 pathways in 3 categories (cellular component, molecular function, and biological processes) of GO and KEGG.

### Prediction of potential miRNA

Target miRNAs of hub genes were predicted using TargetScan (http://www.targetscan.org), miRDB (https://mirdb.org/), and miRWalk (http://mirwalk.umm.uni-heidelberg.de/) databases. The predicted miRNAs were then intersected with the DEmiRNA in GSE95196 to build mRNA-miRNA regulatory network.

### Animals

A total of 33 healthy 4-week-old (n=6), 16-week-old (n=6), and 12-month-old (n=21) C57BL/6 male mice were bought from Shanghai Jihui Laboratory Animal Care Cooperation (China) and kept in the Animal Experimental Center of the Naval Medical University (SYXK 2017-0004). The mice were housed under specific pathogen-free (SPF) conditions with an appropriate environment (20±5°C, 55±5% humidity and 12-h light/dark cycle). The mice were fed with standard chow (corn (40%), bran (25%), bean cake (30%), and others (5%), including salt, bone powder, and necessary vitamins) and had free access to sterile water. All experiments were approved by the Ethics Committee on Animal Experiments of the Naval Medical University (2019CZJS205).

### Isolation of bone marrow ECs (BMECs)

The procedures for the isolation of BMECs were reported previously (19). We euthanized and collected tissue samples from 6 mice of each of the 3 groups. Briefly, the bone marrow cells isolated from mouse femurs, and tibias were cultured with normal culture medium consisting of DMEM (Cytiva, USA) with 10% FBS (Thermo Fisher Scientific, USA) and 1% penicillin/streptomycin (Yuanye, China) overnight. Then, the adherent cells were detached with Accutase (ICT, USA) and BMECs selected using CD31 microbeads for real-time quantitative polymerase chain reaction (RT-qPCR).

### RT-qPCR

Total RNA of cells was extracted using an RNA-Quick Purification Kit (China). Complementary DNA (cDNA) of mRNAs was synthesized using HiScript III RT SuperMix (Vazyme, China). cDNA of miRNAs was synthesized using a miRNA first strand synthesis kit (Agilent Technologies, USA). Transcriptional levels of *Adamts1*, *Adamts2*, *Adamts4*, *Adamts14*, *Col5a1*, *Col5a2*, miR-446i-3p, miR-446i-5p, miR-7218-5p, and *Gapdh* were then measured by RT-qPCR using Taq Pro Universal SYBR qPCR Master Mix (Vazyme). The assays were then performed with the 7900HT Detection System (Applied Biosystems, USA), and the quantification of the mRNA levels was determined using the 2^-ΔΔCT^ method.

### Treatment of AMO-miR-466i-5p *in vivo*

The anti-miRNA oligodeoxyribonucleotide (AMO)-negative control (NC) and AMO-miR-466i-5p were synthesized by GenePharma Co., Ltd. (China) and dissolved in saline at the concentration of 35 mg/mL. *In vivo* experiments were performed following the manufacturer's instructions. After searching the literature, it was found that 80 mg/kg AMO-miRNA is able to treat bone diseases (20,21). Fifteen healthy 12-month-old male C57BL/6J mice were randomly divided into 3 groups (n=5 per group): the control group (saline), AMO-NC group (administered 80 mg/kg AMO-NC), and AMO-miR-466i-5p group (administered 80 mg/kg AMO-miR-466i-5p). Mice were treated with saline and AMO-miR through a tail intravenous injection every 3 days for 6 weeks. Then, the animals were euthanized, and the femurs were collected.

### Micro-computed tomography (**μ**CT)

μCT (Skyscan 1172, Bruker, Belgium) was used to assess the bone mass and structure of the distal femur. The samples were scanned under X-ray source of 80 kV and 124 μA, with resolution of 8 μm. Parameters including trabecular bone volume/total volume (BV/TV, %), trabecular number (Tb.N, g/cc), and cortical thickness (Ct.Th, mm) were obtained.

### Double calcein staining

Mice were administered an intraperitoneal injection of a 0.1% calcein solution (Sigma-Aldrich, catalog number 154071-48-4, USA) at 10 and 3 days prior to the humane euthanasia. Subsequently, the bone tissue sections were subjected to imaging using a fluorescence microscope (Olympus BX61, Japan), and the assessment of periosteal bone and trabecular bone formation was conducted following established methodologies (22).

### Statistical analysis

Data are reported as means±SD. Shapiro-Wilk analysis was carried out to test whether the data had normal distribution. Then, the variances of the two groups were compared by the F-test. The comparison of the average of the two groups was then performed by two-tailed unpaired Student's *t*-test. Significant differences between multiple groups were determined by ANOVA or ANOVA with Sidak's multiple comparisons test. All statistical analyses were carried out by GraphPad prism version 8.0 (Graphpad Software, USA) and R software (https://www.r-project.org/). Differences were considered significant at P<0.05.

## Results

### Identification and functional analysis of the DEGs between young and adult mouse femoral ECs

**Figure 1:**
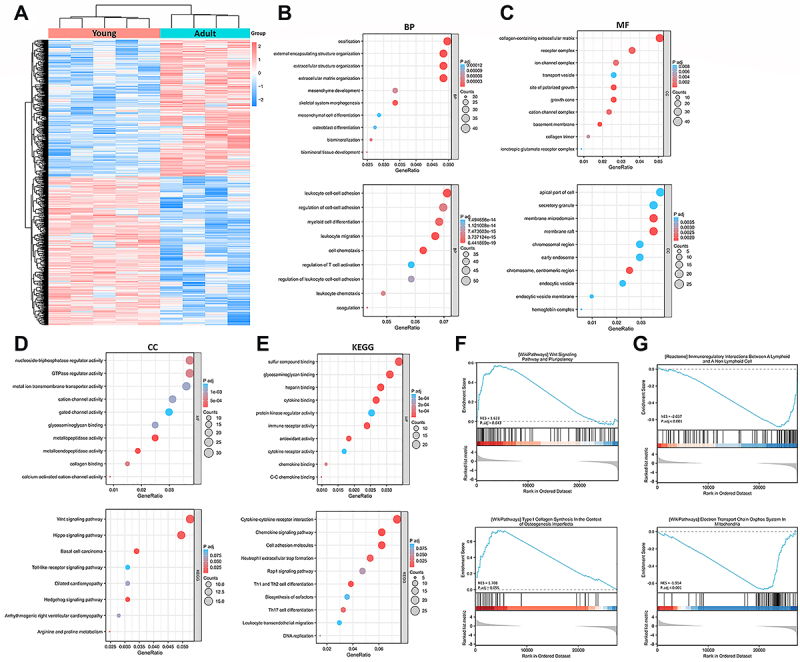
Screening and functional enrichment of differentially expressed genes (DEGs) in capillaries from the ossification front of the juvenile mouse samples compared to adult samples. **A**, Heatmap of the DEGs identified in GSE148804 dataset. **B**, Gene Ontology (GO) annotation (biological processes, BP) of up-regulated and down-regulated DEGs. **C**, GO annotation (molecular function, MF) of up-regulated and down-regulated DEGs. **D**, GO annotation (cellular component, CC) of up-regulated and down-regulated DEGs. **E**, Kyoto Encyclopedia of Genes and Genomes (KEGG) annotation of up-regulated and down-regulated DEGs. **F**, Representative up-regulated pathways analyzed by gene set enrichment analysis (GSEA). **G**, Representative down-regulated pathways analyzed by GSEA.

A total of 1626 DEGs were identified, comprising 845 up-regulated genes and 781 down-regulated genes (young *vs* adult) (Figure 1A). Gene ontology (GO) enrichment analysis revealed a strong association between the up-regulated genes and processes related to bone formation, including ossification, collagen-containing extracellular matrix, and nucleoside-triphosphatase regulator activity (Figure 1B-D). Consistent with these findings, Kyoto Encyclopedia of Genes and Genomes (KEGG) analysis indicated the activation of the Wnt and Hippo signaling pathways, which are known to promote osteogenesis and biomineralization, in young ECs (Figure 1E). In contrast, the down-regulated GO terms were predominantly linked to immune regulation, encompassing leukocyte cell-cell adhesion, myeloid cell differentiation, secretory granule formation, and cytokine binding (Figure 1B-D). KEGG analysis further demonstrated the suppression of pathways associated with cytokine-cytokine receptor interaction, chemokine signaling, and Rap1 signaling in the juvenile samples (Figure 1E). Furthermore, the representative pathways identified through gene set enrichment analysis (GSEA) are depicted in Figure 1F and G. Notably, the Wnt pathway and Type I synthesis were up-regulated, whereas immunoregulatory interactions and electron transport chain oxidative phosphorylation system were down-regulated.

### PPI analysis and hub gene identification of the DEGs between young and adult mouse type H vessel

**Figure 2:**
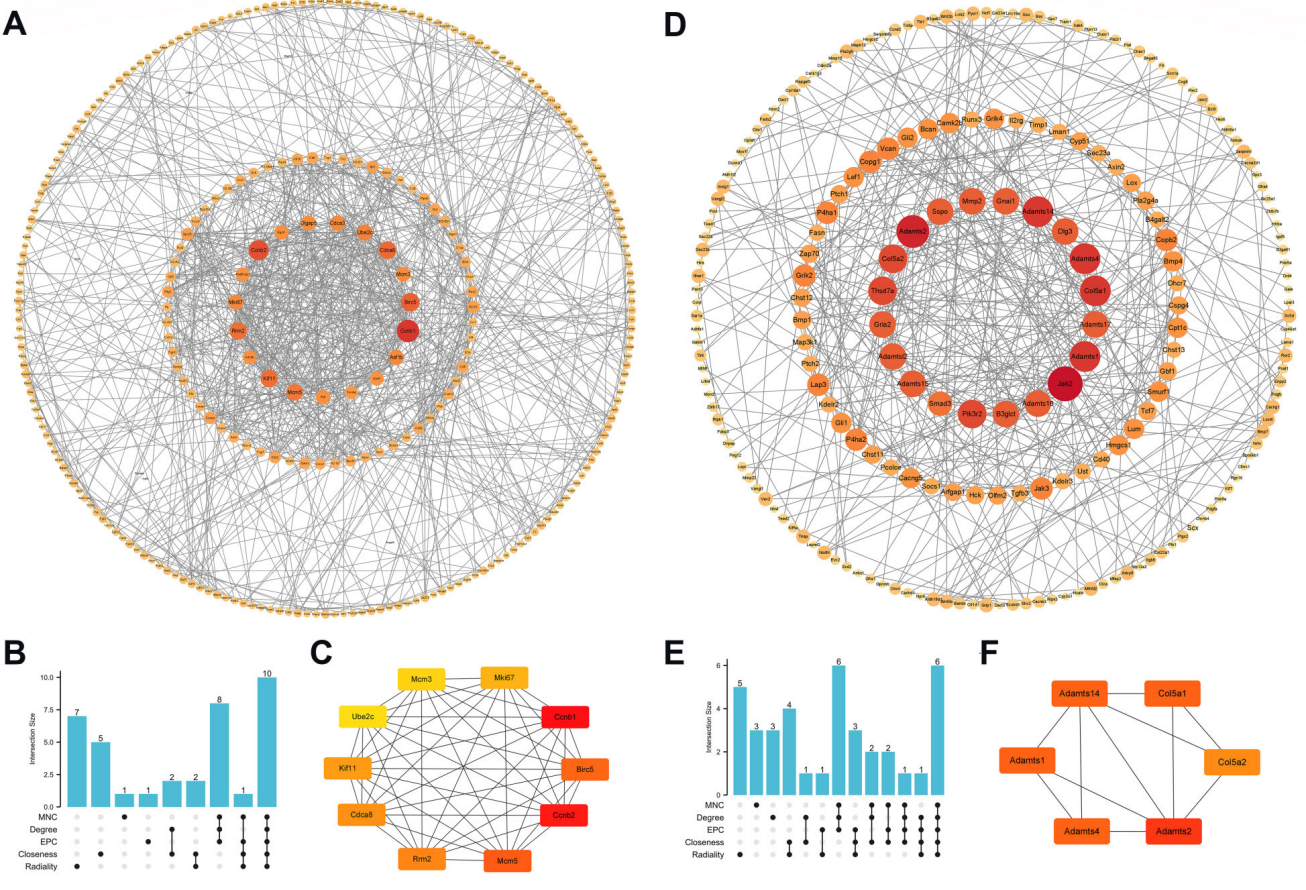
Protein-protein interaction (PPI) network construction and hub gene screening of differentially expressed genes (DEGs) in GSE148804 dataset. **A**, PPI network constructed with the shared up-regulated DEGs. **B**, Hub genes were identified by intersection of top 10 up-regulated genes from 5 algorithms including MNC, Degree, EPC, Closeness, and Radiality. **C**, PPI network diagram of the ten hub up-regulated genes. **D**, PPI network constructed with the shared down-regulated DEGs. **E**, Hub genes were identified by intersection of top 10 down-regulated genes from 5 algorithms including MNC, Degree, EPC, Closeness, and Radiality. **F**, The PPI network diagram of the six hub down-regulated genes.

In order to elucidate the interactions among the differentially expressed genes (DEGs), protein-protein interaction (PPI) networks were constructed separately for the up-regulated and down-regulated gene sets. Subsequently, we employed five algorithms, namely MNC, Degree, EPC, Closeness, and Radiality, available in the Cytohubba plugin within the Cytoscape software (https://cytoscape.org/), to identify key genes (Figure 2). Notably, the top 10 genes identified by all five algorithms were considered as pivotal candidates. Among the up-regulated genes, the following ten genes emerged as significant hub genes: *Ccnb1*, *Ccnb2*, *Mcm3*, *Mcm5*, *Birc5*, *Mki67*, *Rrm2*, *Cdca8*, *Kif11*, and *Ube2c*. Similarly, the analysis identified six down-regulated hub genes: *Adamts1*, *Adamts2*, *Adamts4*, *Adamts14*, *Col5a1*, and *Col5a2* (Figure 2).

### Target miRNAs prediction for hub genes

**Figure 3:**
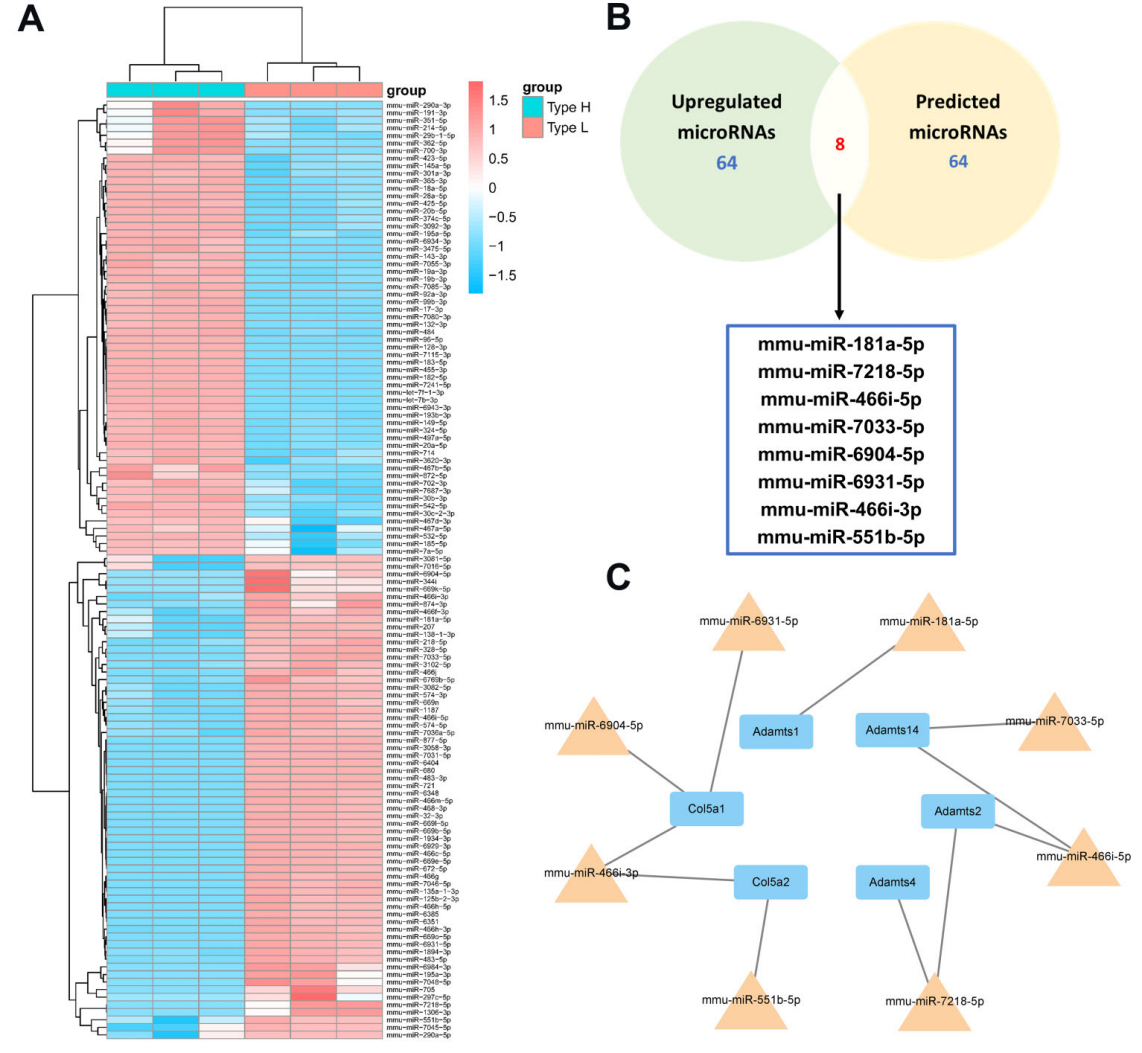
Construction of miRNA-mRNA regulatory network. **A**, Heatmap of the differentially expressed miRNAs (DEmiRNAs) between type H and type L epithelial cells identified in the GSE95196 dataset. **B**, Venn diagram showing the numbers of DEmiRNAs and predicted miRNAs in TargetScan, miRDB, and miRWalk databases. **C**, Relationship between 6 hub genes and 8 selected miRNAs (miRNA-mRNA regulatory network).

To investigate potential determinants underlying the observed changes in gene expression, we employed the GSE95196 dataset, which encompassed three type-H and three type-L vessel samples, to conduct an analysis targeting miRNAs potentially associated with the modulation of hub genes. A total of 124 differentially expressed miRNAs (DEmiRNAs) were identified, comprising 64 up-regulated and 60 down-regulated miRNAs specifically in type L ECs (Figure 3A). Furthermore, by leveraging TargetScan, miRDB, and miRWalk online databases, we successfully identified 64 putative target miRNAs for the six hub genes (Figure 3B). Through an intersection analysis of the aforementioned target miRNAs and the up-regulated miRNAs, we discerned the presence of eight miRNAs (Figure 3B). Subsequently, a regulatory network encompassing the eight identified miRNAs and the six hub genes was constructed based on the predicted miRNA-RNA interactions (Figure 3C).

### Validation of the hub genes and related miRNAs *in vivo*

**Figure 4:**
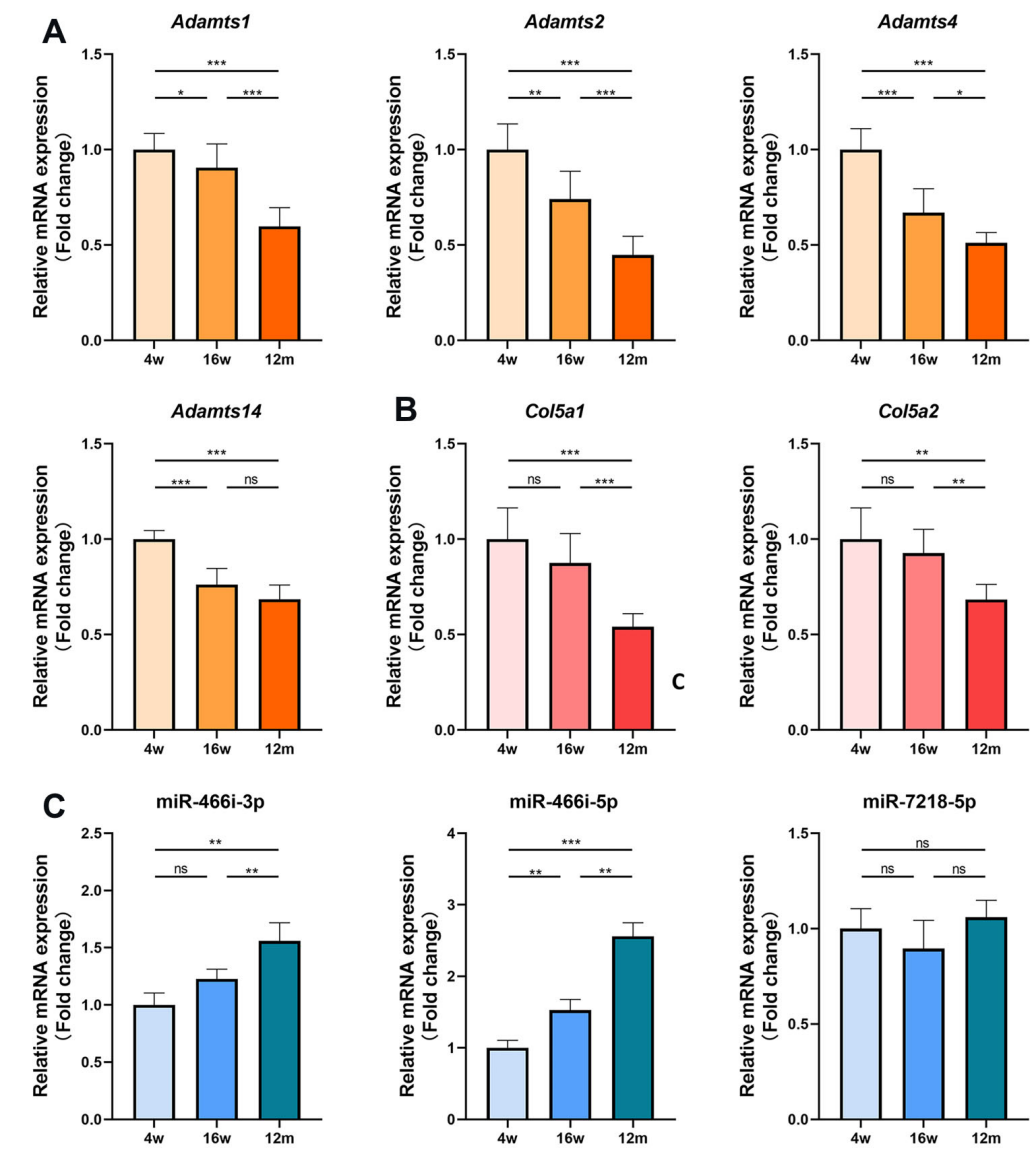
Validation of the hub genes and miRNAs *in vivo* in age groups of 4 and 16 weeks, and 12 months. The transcriptional expression levels of **A**) Adamts family genes (*Adamts1*, *Adamts2*, *Adamts4*, *Adamts14*), **B**) Col5a family genes (*Col5a1*, *Col5a2*), and **C**) miRNAs (miR-466i-3p, miR-466i-5p, miR-7218-5p). Data are reported as means±SD for n=6 mice per group in all panels. *P<0.05; **P<0.01; ***P<0.001 (ANOVA). ns: not significant.

To validate the significance of the identified hub genes in an *in vivo* context, we performed RT-qPCR assays to assess their expression levels in ECs derived from young (4-week-old), adult (16-week-old), and old (12-month-old) mice. Our findings revealed a gradual decline in the transcriptional levels of *Adamts1*, *Adamts2*, and *Adamts4* as age advanced (Figure 4A and B). Furthermore, a notable discrepancy in the expression of *Adamts14* was observed between young and adult ECs, while its expression remained comparable between adult and old samples (Figure 4A). Conversely, no substantial alterations were detected in the expression of *Adamts1* and *Adamts4* between the young and adult groups, but a significant decrease was observed between adult and old samples (Figure 4A). In order to shed further light on the functional relevance of the associated miRNAs, we examined the expression of three miRNAs (miR-466i-3p, miR-466i-5p, and miR-7218) known to target two of the central genes (Figure 4C). Our results exhibited an inverse correlation between the expression levels of miR-466i-5p and age. Additionally, miR-466i-3p did not display significant changes between the 4-week-old and 16-week-old groups, but exhibited a significant increase between the 16-week-old and 12-month-old groups, while the expression of miR-7218 did not demonstrate significant variations.

### Inhibition of miR-466i-5p improved age-related bone loss *in vivo*

Given the considerable alteration observed in the expression level of miR-466i-5p as age advances, we sought to investigate its therapeutic potential by administering AMO of miR-466i-5p to 12-month-old mice. Given that AMOs are primarily metabolized by the liver and excreted by the kidneys, we assessed the hepatic and renal functions following a 6-week administration of AMO. Histological examination utilizing H&E staining revealed no significant differences among the three groups (Supplementary Figure S1A and B). Furthermore, the serum markers for liver function (alanine transaminase (ALT), aspartate transaminase (AST)), and kidney function (blood urea nitrogen (BUN)) did not demonstrate significant alterations after AMO treatment, thereby indicating the absence of substantial toxicity associated with AMO (Supplementary Figure S1C and D).

**Figure 5:**
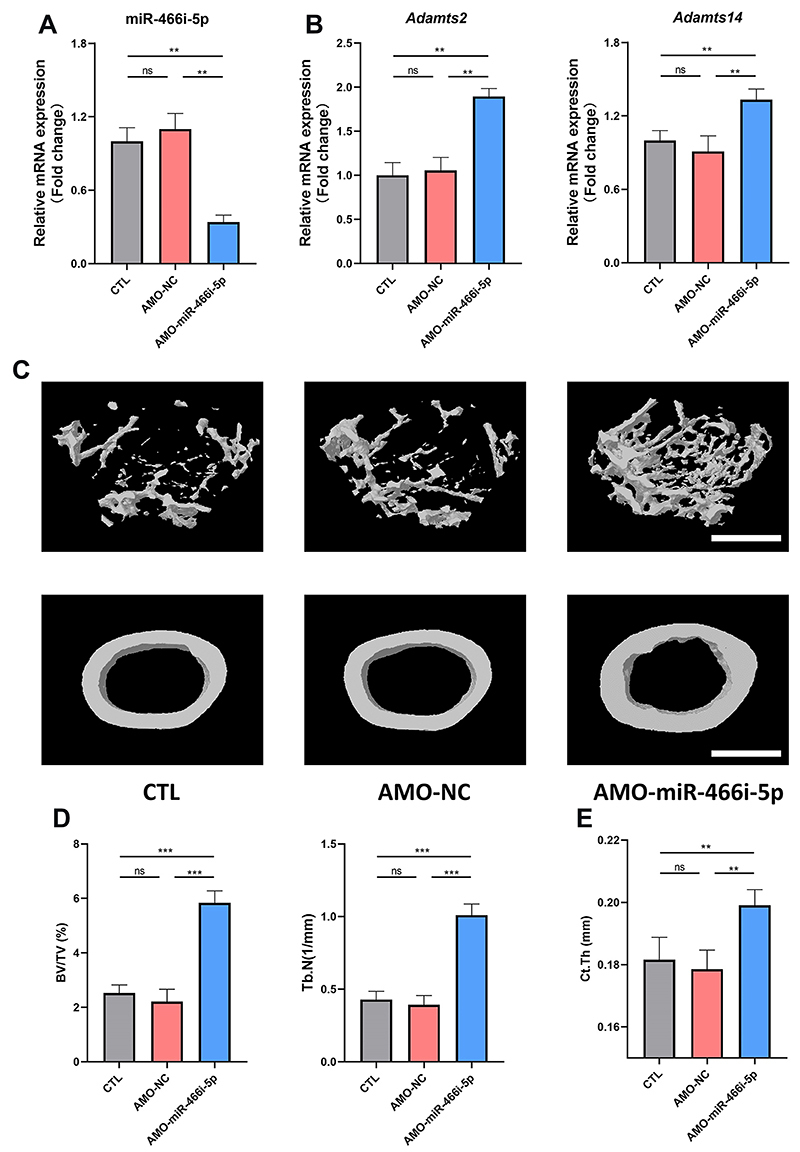
The treatment of anti-miRNA oligodeoxyribonucleotide (AMO)-miR-466i-5p alleviates age-related bone loss. **A**, mRNA expression level of miR-466i-5p. **B**, mRNA expression level of *Adamts2* and *Adamts14*. **C**, Representative micro CT analysis of trabecular and cortical bone of the distal femur. Scale bar: 1 mm. **D**, Calculations of trabecular parameters including bone volume/total volume (BV/TV) and trabecular number (Tb.N). **E**, Calculations of trabecular thickness (Ct.Th). Data are reported as means±SD for n=6 mice per group in all panels. **P<0.01; ***P<0.001 (ANOVA). ns: not significant. CTL: control; NC: negative control.

Through RT-qPCR analysis, we confirmed a significant decrease in miR-466i-5p expression levels in ECs, accompanied by an enhancement in the levels of *Adamts2* and *Adamts14* upon AMO-miR-466i-5p treatment (Figure 5A and B). Subsequently, micro CT imaging was performed to evaluate changes in trabecular and cortical bone mass (Figure 5C-E). Quantitative analysis revealed significant improvements in BV/TV, Tb.N, and Ct.Th following administration of AMO-miR-466i-5p. In addition, double calcein staining showed that AMO-miR-466i-5p increased mineral apposition rate (MAR) and bone formation rate per unit of bone surface (BFR/BS) (Supplementary Figure S2). These results demonstrated the potential of miR-466i-5p as a therapeutic intervention for age-related bone loss by targeting bone marrow ECs.

## Discussion

The pathogenesis of age-related osteoporosis is concomitant with changes in vascular ECs (1,23). Type H ECs, characterized by their heightened expression levels of CD31 and EMCN, reside in close proximity to skeletal stem cells and osteoprogenitor cells (24). Moreover, CD31^hi^Emcn^hi^ ECs manifest notable upregulation of osteogenic factors, including platelet-derived growth factor A (PDGF-A), fibroblast growth factor 1 (FGF1), and members of the bone morphogenetic protein (BMP) family, namely BMP1, BMP4, and BMP6 (25). In contrast, sinusoidal type L ECs actively engage in the orchestration of hematopoiesis and myelopoiesis (26). As age advances, the population of type H ECs experiences a decline, consequently culminating in the gradual accumulation of cortical and trabecular bone loss, ultimately causing the onset of osteoporosis (3). Our findings further indicated a significant enrichment of osteogenic pathways in juvenile femoral ECs, whereas factors associated with immune regulation exhibit an upregulated expression profile in adult specimens. This observed phenomenon can plausibly be attributed to the transition from type H to type L ECs.

Subsequently, we discerned pivotal genes that displayed enrichment in youthful vascular endothelial cells (ECs), encompassing four members of the Adamts gene family (*Adamts1*, *Adamts2*, *Adamts4*, and *Adamts14*), as well as two members of the Col5a gene family (*Col5a1* and *Col5a2*). The ADAMTS proteins are secretory zinc endopeptidases that regulate the intricate organization of the extracellular matrix (ECM) (27). Mutations in *ADAMTS2*, *ADAMTS4*, *ADAMTS10*, and *ADAMTS17* have been linked to skeletal anomalies characterized by the manifestation of shortened hands and fingers, underscoring the critical role of this enzyme family in bone metabolism (27,28). Type V collagen, translated by the *Col5a1* and *Col5a2* genes, controls the assembly of fibrils and contributes to the composition of the bone matrix (29,30). Disruptions in *Col5a1* have been implicated in the pathogenesis of osteogenesis imperfecta (31,32). Our investigation revealed an inverse relationship between the transcriptional levels of *Adamts1*, *Adamts2*, *Adamts4*, *Adamts14*, *Col5a1*, and *Col5a2* in femoral ECs and advancing age, thereby confirming their involvement in the age-related deterioration of the bone vascular network.

The regulation of skeletal ECs relies on miRNAs. In a study by Yang et al. (17), it was reported that the miR-497-195 cluster plays a critical role in maintaining endothelial Notch activity and HIF-1a stability, thereby promoting angiogenesis in CD31^hi^Emcn^hi^ ECs and facilitating coupled osteogenesis. Additionally, Wang et al. (33) demonstrated that miR-143 exhibits high expression levels in type H endothelium and positively regulates bone formation. These investigations underscore the pivotal regulatory role of miRNAs in the process of skeletal vascularization. In light of these findings, we identified miRNAs that exhibited significant downregulation in type H ECs compared to type L ECs. Subsequently, we constructed a comprehensive miRNA-mRNA interaction network based on predicted relationships between differentially expressed miRNAs and hub genes. Notably, the expression level of miRNA-466i-5p displayed a substantial increase with age, and *in vivo* studies provided evidence that the inhibition of miRNA-466i-5p through anti-miRNA oligonucleotides effectively mitigated age-related trabecular and cortical bone loss, highlighting its potential as a promising therapeutic target.

### Conclusion

Collectively, our findings serve to highlight the inherent and paramount role played by skeletal ECs in governing bone metabolism, firmly establishing them as promising therapeutic targets for the efficacious management of osteoporosis.

## Supplementary Material

Click here to view [pdf].
