# Supplementary material for: Integrative analyses of genetic characteristics associated with skeletal endothelial cells
Source: Braz J Med Biol Res. 2024 Apr 19;57:e13339. doi: 10.1590/1414-431X2024e13339 (PMC11027181; doi:10.1590/1414-431X2024e13339)
Supplement: Supplementary file 1 [file 1414-431X-bjmbr-57-e13339-suppl.pdf]

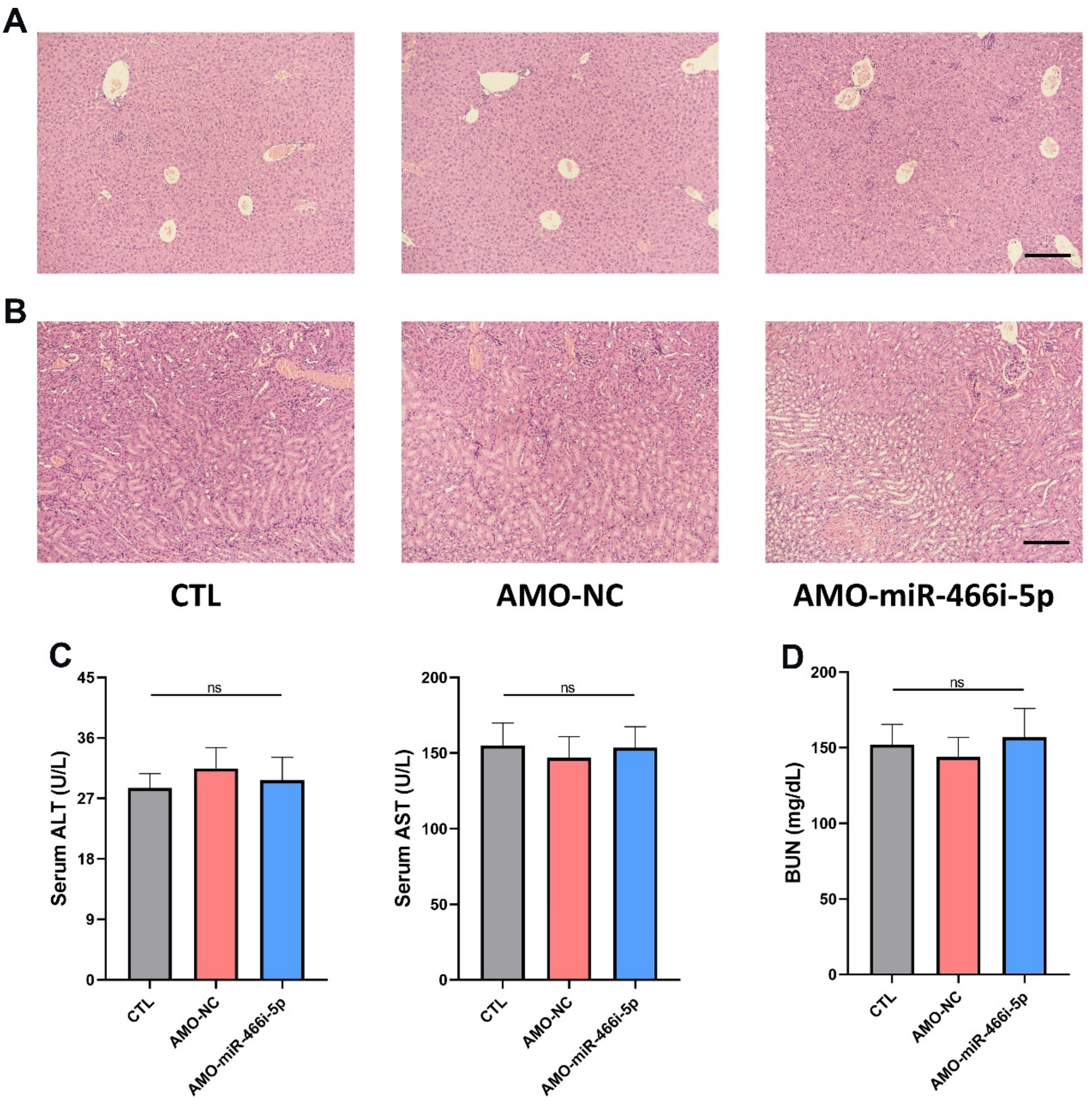

Figure S1. The administration of AMO-miR caused no significant toxicity. The mice were sacrificed after 6 weeks of saline, AMO-NC, or AMO-miR-466i-5p administration. Representative HE staining of liver (**A**) and kidney (**B**). Scale bar: 250 μm. **C**, Serum levels of alanine transaminase (ALT) and aspartate transferase (AST) and **D**, blood urea nitrogen (BUN). ns: non-significant.

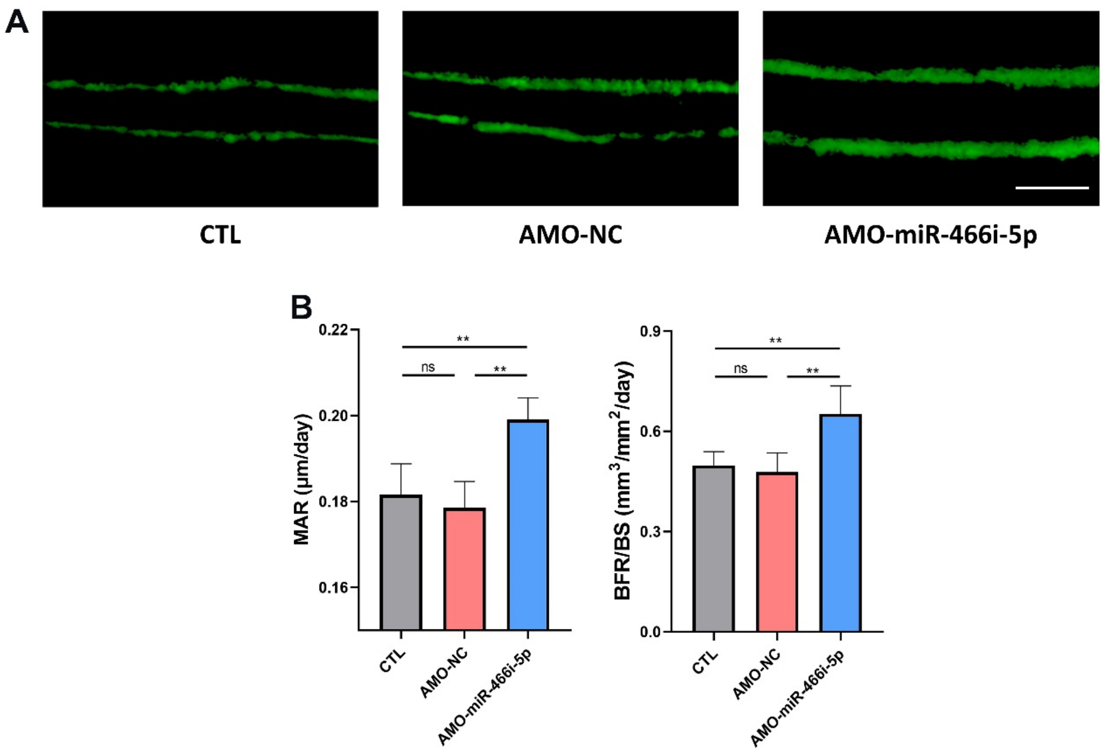

Figure S2. The administration of AMO-miR-466i-5p promotes bone formation. **A**, Representative figures of double calcein staining. **B**, Quantification of mineral apposition rate (MAR) and bone formation rate per unit of bone surface (BFR/BS). **P<0.01 (ANOVA). ns: non-significant.
